# A Randomised, Double-Blind, Controlled Vaccine Efficacy Trial of DNA/MVA ME-TRAP Against Malaria Infection in Gambian Adults

**DOI:** 10.1371/journal.pmed.0010033

**Published:** 2004-10-26

**Authors:** Vasee S Moorthy, Egeruan B Imoukhuede, Paul Milligan, Kalifa Bojang, Sheila Keating, Pauline Kaye, Margaret Pinder, Sarah C Gilbert, Gijs Walraven, Brian M Greenwood, Adrian S. V Hill

**Affiliations:** **1**Medical Research Council LaboratoriesBanjulGambia; **2**Centre for Clinical Vaccinology and Tropical Medicine, Nuffield Department of Clinical Medicine, University of Oxford, Churchill HospitalOxfordUnited Kingdom; **3**Wellcome Trust Centre for Human Genetics, University of OxfordOxfordUnited Kingdom; **4**London School of Hygiene and Tropical MedicineLondonUnited Kingdom; Kenya Medical Research InstituteKenya

## Abstract

**Background:**

Many malaria vaccines are currently in development, although very few have been evaluated for efficacy in the field. Plasmodium falciparum multiple epitope (ME)– thrombospondin-related adhesion protein (TRAP) candidate vaccines are designed to potently induce effector T cells and so are a departure from earlier malaria vaccines evaluated in the field in terms of their mechanism of action. ME-TRAP vaccines encode a polyepitope string and the TRAP sporozoite antigen. Two vaccine vectors encoding ME-TRAP, plasmid DNA and modified vaccinia virus Ankara (MVA), when used sequentially in a prime-boost immunisation regime, induce high frequencies of effector T cells and partial protection, manifest as delay in time to parasitaemia, in a clinical challenge model.

**Methods and Findings:**

A total of 372 Gambian men aged 15–45 y were randomised to receive either DNA ME-TRAP followed by MVA ME-TRAP or rabies vaccine (control). Of these men, 296 received three doses of vaccine timed to coincide with the beginning of the transmission season (141 in the DNA/MVA group and 155 in the rabies group) and were followed up. Volunteers were given sulphadoxine/pyrimethamine 2 wk before the final vaccination. Blood smears were collected weekly for 11 wk and whenever a volunteer developed symptoms compatible with malaria during the transmission season. The primary endpoint was time to first infection with asexual P. falciparum. Analysis was per protocol.

DNA ME-TRAP and MVA ME-TRAP were safe and well-tolerated. Effector T cell responses to a non-vaccine strain of TRAP were 50-fold higher postvaccination in the malaria vaccine group than in the rabies vaccine group. Vaccine efficacy, adjusted for confounding factors, was 10.3% (95% confidence interval, −22% to +34%; *p* = 0.49). Incidence of malaria infection decreased with increasing age and was associated with ethnicity.

**Conclusions:**

DNA/MVA heterologous prime-boost vaccination is safe and highly immunogenic for effector T cell induction in a malaria-endemic area. But despite having produced a substantial reduction in liver-stage parasites in challenge studies of non-immune volunteers, this first generation T cell–inducing vaccine was ineffective at reducing the natural infection rate in semi-immune African adults.

## Abstract 2

Inducing an immune response is just one part of what a vaccine needs to do before it will protect against episodes of malaria.

## Introduction

The disease burden of malaria has increased in recent years partly because of the rise of drug-resistant Plasmodium falciparum parasites [1] and insecticide-resistant *Anopheline* spp. vectors [2]. There is an urgent need for effective malaria control methods to reduce mortality and morbidity from malaria in endemic countries. Detailed analysis of immunological mechanisms of immunity against malaria in humans and experimental animals has indicated a likely protective role for T cell responses against the liver stages of P. falciparum [3,4,5,6,7,8,9]. Comparison of a variety of means of immunisation to induce protective T cell responses in animal models has identified heterologous prime-boost immunisation, i.e., sequential immunisation with two different vaccines with the same recombinant DNA sequence, as a particularly effective approach [10,11]. DNA and viral vaccines recombinant for a malarial DNA sequence known as multiple epitope (ME)–thrombospondin-related adhesion protein (TRAP), which were designed to induce protective immunogenicity against liver-stage P. falciparum malaria, were manufactured to explore this approach [12]. γ-interferon T cell responses to ME and TRAP peptides were associated with protection from severe malarial anaemia in a prospective study of Kenyan children [13].

DNA and modified vaccinia virus Ankara (MVA)'s excellent safety profiles in malaria-naïve and semi-immune volunteers have been discussed previously [12]. In several studies, prime-boost immunisation (usually with DNA/MVA) has been highly immunogenic for CD4+ and CD8+ T cell induction against infectious pathogens and cancers in both murine and nonhuman primate studies [14,15,16,17,18,19]. DNA/MVA vaccination was protective 7 mo after vaccination in an HIV macaque model [20].

Priming with three 2-mg intramuscular DNA ME-TRAP vaccinations at 3-wk intervals, followed by boosting with one intradermal MVA ME-TRAP vaccination of 1.5 × 10^8^ plaque-forming units, produced very strong vaccine-induced CD4+ and CD8+ T cell responses in previous phase I studies in the United Kingdom [21]. The immunogenicity of two DNA ME-TRAP primes followed by one MVA ME-TRAP boost at these doses is similarly high in both the United Kingdom (S. Dunachie and A. V. S. Hill, unpublished data) and Gambia [22]. DNA ME-TRAP/MVA ME-TRAP regimens led to a delay in time to parasitaemia compared to unvaccinated controls after high-dose heterologous sporozoite challenge of malaria-naïve individuals [21].

To follow up these encouraging findings in volunteers, we have conducted a randomised, controlled trial of DNA ME-TRAP/MVA ME-TRAP in a rural part of Gambia to explore whether this vaccine combination could provide protection against natural P. falciparum infection. We chose a two-DNA prime, one-MVA boost regimen with 3-wk between doses because this is a three-dose regimen that would be amenable to integration with the World Health Organization/United Nations Children's Fund Expanded Program on Immunization, with the necessary supporting safety and immunogenicity data both from adults in the United Kingdom and Gambia. We used 3-wk intervals because 4-wk intervals had not been evaluated in phase I trials previously, hence bridging studies would be necessary to bridge to the three-dose, 4-wk interval Expanded Program on Immunization schedule.

## Methods

### Vaccines

The malarial DNA sequence is known as ME-TRAP. The ME string contains 14 CD8+ T cell epitopes, one CD4+ T cell epitope, and two B cell epitopes from six pre-erythrocytic P. falciparum antigens. It also contains two non-malarial CD4+ T cell epitopes [23]. The ME string is fused in frame to the entire T9/96 strain of P. falciparum TRAP [10,24,25]. The individual epitopes making up the ME string are described in detail elsewhere [23]. The strain of P. falciparum used to produce the vaccine construct is T9/96. The candidate malaria vaccines were manufactured to Good Manufacturing Practice by contract manufacturers (DNA ME-TRAP by Qiagen, Hilden, Germany; MVA ME-TRAP by IDT, Rosslau, Germany). DNA ME-TRAP was supplied as a single dose of2 mg in 2-ml vials. MVA ME-TRAP was supplied as two-dose vials, each containing 3 × 10^8^ plaque-forming units in 0.8 ml. The rabies vaccine (Chiron Behring, Marburg, Germany) was supplied as a lyophilised single-dose vial with accompanying diluent and syringe. This vaccine was chosen because of its public health benefit in Gambia.

### Study Setting and Volunteers

Approval was obtained from the Joint Gambian Government/Medical Research Council Ethics Committee, the Oxford Tropical Research Ethics Committee, and the London School of Hygiene and Tropical Medicine Ethics Committee. An independent Data and Safety Monitoring Board provided oversight for the trial. In addition, independent clinical trial monitors monitored the trial for adherence to International Committee on Harmonisation Good Clinical Practice guidelines.

Malaria incidence is highly seasonal in Gambia. The climate is typical of sub-Sahelian Africa, with a long dry season followed by a relatively short rainy season from July to October. Rainfall averages about 600 mm per year. Morbidity and mortality from malaria both occur more frequently during the rainy season. However, in 2002, Gambia experienced a drought, and the malaria season was delayed, with few disease episodes before October. The entomological inoculation rate varies between less than 1 and greater than 100 in Gambia [26]. Based on previous years' data, we assumed that the average cumulative incidence over a number of years would be about 60% (interquartile range [IQR], 50%–70%), with very few years with incidence less than 40% or more than 90%. Allowing for 15% loss to follow-up, and using a significance level of 0.05, the median power of a study with 372 participants would be 90% (95% confidence interval [CI], 69%–98%) if the vaccine efficacy were 40%. Volunteers were recruited from 13 villages in the North Bank Division of Gambia in July 2002 with follow-up to December 2002 [26]. The villages were chosen for proximity to the alluvial flood plain. A strong association between proximity to the flood plain and entomological inoculation rate has been seen in this part of Gambia [27], and the entomological inoculation rate in the study area is likely to have been in the range of 10–20 infectious bites per year.

Before recruitment, meetings were held with village heads and elders, followed by general village meetings at which the study was explained. Volunteers received information sheets and consent forms translated into the three local languages in Arabic script, as well as in English. After written informed consent was obtained by a study physician, age and identity were checked, pre-test HIV counselling occurred, and potential volunteers underwent clinical evaluation, including a full medical history and clinical examination. They were screened for haematological (full blood count), renal (plasma creatinine), and hepatic (plasma alanine aminotransferase [ALT]) dysfunction, and duplicate malaria smears were made. Exclusion criteria included any chronic illness detected by clinical evaluation, ALT greater than 42 (international units/litre), creatinine greater than 130 (micromoles/litre), packed cell volume less than 30%, positive antibody ELISA to HIV-1 or HIV-2, simultaneous participation in another clinical trial, blood transfusion in the month prior to vaccination, previous experimental malaria vaccination, administration of another vaccine within 2 wk of vaccination, previous rabies vaccination, allergy to any previous vaccine or to sulphadoxine/pyrimethamine, history of splenectomy, and any treatment with immunosuppressive drugs. Eligible volunteers were given a unique study number and a photographic identity card. Parental written informed consent was obtained for volunteers aged 15–17 y.

### Procedures

A member of the Data and Safety Monitoring Board generated and held the randomisation code that associated each study number with a specific vaccine. A blocking procedure was used, and whole villages were enrolled with sequential study numbers to ensure balanced numbers in each group. During the course of the study investigators and volunteers did not know the size of the blocks, nor were they aware of which vaccine preparation was administered to a particular volunteer. Opaque sealed envelopes were used for vaccine allocation. Study numbers were not preprinted on vials but were written on vials at vaccination. Used vials were checked for correct allocation off-site. Vaccination was performed by nurses who played no other part in the trial.

Volunteers were randomly assigned to receive either (1) two 2-mg doses of DNA ME-TRAP followed by a single 1.5 × 10^8^-plaque-forming-units dose of MVA ME-TRAP or (2) three doses of rabies vaccine; injections were given on days 0, 21, and 42, timed to coincide with the start of the rainy season. The first two doses of vaccine consisted of two intramuscular injections, one into each deltoid muscle. DNA ME-TRAP was given as 1 ml and rabies vaccine as 0.5 ml into each arm. The third dose of vaccine was given as four intradermal injections into the skin overlying the deltoid muscle, with two injections into each arm. The malaria vaccine group received MVA ME-TRAP as four 0.1-ml injections, whereas the control group received four 0.05-ml injections of rabies vaccine. Two weeks before administration of the third dose, all volunteers received three tablets of sulphadoxine/pyrimethamine to clear blood-stage P. falciparum infections [23].

After each vaccination volunteers were observed for 1 h and visited at home on the first, second, and seventh day postvaccination for assessment of local adverse events (discolouration, induration, blister formation, pain, or limitation of arm motion), systemic adverse events (headache, nausea, malaise, or elevated axillary temperature), and unsolicited adverse events. One week and 13 wk after the third vaccination, venous blood was collected for repeated measurement of full blood count, ALT, and creatinine.

Since vaccination with MVA causes a characteristic local reactogenicity in some subjects, specific steps were taken to ensure that the participants were evaluated in a double-blinded manner. Field workers who assessed reactogenicity after the third dose were different from those who undertook surveillance during the parasitological follow-up period. During the surveillance period, starting 2 wk after the third dose of vaccine, volunteers were visited twice weekly and asked whether they had attended a health centre. At weekly visits blood smears and axillary temperatures were taken. At midweek visits, blood smears and temperature were taken if symptoms compatible with malaria were present. Investigators and field supervisors made random visits to ensure accurate data collection. This active case detection was supplemented by passive case detection by study nurses to whom volunteers had 24-h access at three of the study villages and by a clinic at Medical Research Council Farafenni (20 km from the study villages). Symptomatic malaria was defined as the presence of asexual P. falciparum parasites at any parasitaemia with either an axillary temperature of 37.5 °C or more or one or more of the following symptoms: headache, myalgia, arthralgia, malaise, nausea, dizziness, or abdominal pain.

When blood smears were obtained, two sets of duplicate blood smears (four smears in total) were made. A Field's stain was performed on films obtained from subjects with possible clinical malaria and the films read immediately. Two further smears (“A” and “B” slides) were stained with Giemsa after overnight drying; 100 high-power fields were read by two slide readers before a film was declared negative. The presence of P. falciparum parasites was confirmed by a supervisor before a slide was declared positive. The arithmetic mean of the A and B slides was used to determine parasite density. If parasite densities for A and B slides were markedly discrepant, a third read was performed by the supervisor and this read was used for analysis. Parasite density was expressed per microlitre (assuming one parasite per high power field equals 500 parasites/μl). Full blood counts were performed and packed cell volumes were measured in a CA620 cell analyser (Medonic, Stockholm, Sweden). ALT (international units/litre) and creatinine (micromoles/litre) were measured in a Visual analyser (bioMérieux, Craponne, France).

Effector T cell responses were assessed in ex vivo γ-interferon enzyme-linked immunospot (ELISPOT) assays for 98 volunteers randomly selected from a substudy list containing a 3:1 ratio of participants receiving malaria vaccines to participants receiving rabies (control) vaccines. For this assay, 4 × 10^5^ peripheral blood mononuclear cells (PBMCs) were assayed as described [28], using Millipore (Billerica, Massachusetts, United States) MAIP S45 plates for 18–20 h before being developed. Mabtech (Stockholm, Sweden) antibodies were used, and counting of spots was performed blinded to vaccine allocation with the AutoImmun Diagnostika (Strassberg, Germany) computerised system. All peptides were at 25 μg/ml concentration. A single pool contained all ME peptides. Four pools each were used of 20-mer peptides, overlapping by ten amino acids, to span the entire TRAP antigen of the T9/96 and 3D7 strains of P. falciparum.

### Statistical Analysis

An analysis plan, written before unblinding, specified exclusion criteria, statistical methods, and important covariates (age, village of residence, and bednet use [defined as sleeping nightly under an intact or impregnated bednet]). Ethnic group, though not specified as a covariate in the analysis plan, was found on analysis to be associated with the risk of infection, and was included as a covariate. The primary endpoint was time to first infection with asexual *P. falciparum,* defined as the number of days from the start of the surveillance period to the date of the first positive slide. Vaccine efficacy was calculated from the hazard ratio estimated by Cox's regression, adjusting for the effects of prognostic variables. Volunteers who received fewer than three doses or who were parasitaemic both prevaccination and at the beginning of surveillance without an intervening negative blood smear were excluded from the primary analysis but included in a secondary analysis. Observations on individuals who were lost to follow-up or were missing from trial data for 3 wk were censored. Analyses were done with Stata version 7 (Stata Corporation, College Station, Texas, United States). ELISPOT responses were analysed as follows. After subtraction of medium-alone values from each pooled peptide response, responses were summed across T9/96 and 3D7 pools. Geometric means were calculated for T9/96 TRAP, 3D7 TRAP, and ME string responses. Responses in the two groups were compared using the Mann-Whitney test.

## Results

**Figure 1:**
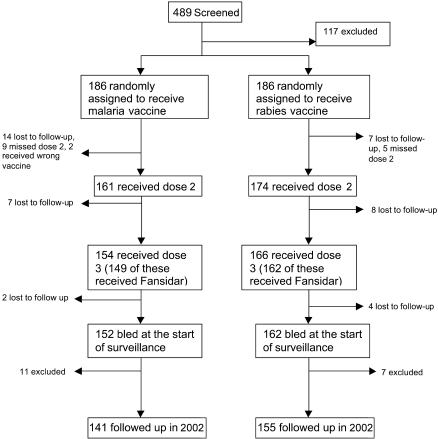
Trial Profile

In total, 489 volunteers were screened (Figure 1), of whom 117 were excluded for the following reasons: 46 could not be found on the day of vaccination, 44 were not eligible (anaemia, ALT, creatinine, HIV, various medical conditions, too young or too old), and 27 withdrew consent. Thus, 372 volunteers aged 15–45 y were enrolled. Of these, 335 men (90%) received their second dose of vaccine, and 320 of these received the third dose. Some 52 volunteers who were randomised did not receive three doses (two men received the wrong vaccine at the second dose, 26 left the study area, 23 withdrew consent, and one was withdrawn because he developed pneumonia between the first and second doses). In total, 296 volunteers (141 in the malaria group and 155 in the rabies group) received three doses and were followed up, 277 of whom completed 11 wk of surveillance. Additional data were available for 14 volunteers who did not receive all three vaccine doses (all 14 received the first and third doses), for two volunteers in the malaria vaccine group who received the wrong vaccine at the second dose, and for 18 volunteers who were parasitaemic both before vaccination and at the start of surveillance. These individuals were included in a secondary analysis. Losses to follow-up were similar in the two groups. Prognostic variables were similarly distributed in the two groups at the start of surveillance (Table 1). This trial is reported in accordance with CONSORT guidelines (Table S1).

**Table 1:**
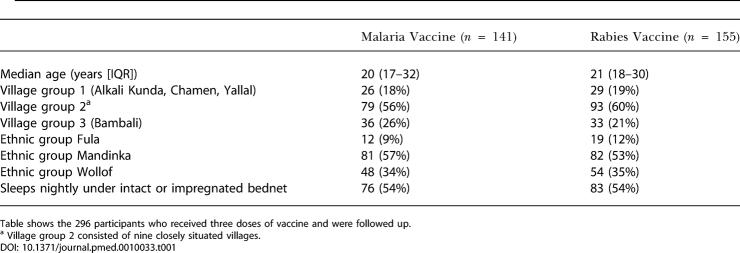
Characteristics of the Trial Cohorts at the Start of Surveillance

Table shows the 296 participants who received three doses of vaccine and were followed up

^a^ Village group 2 consisted of nine closely situated villages

### Vaccine Safety

No clinically significant differences in packed cell volume, ALT, or creatinine were seen in either vaccine group. One volunteer who received rabies vaccine had a history of breathlessness and chest pain several years prior to enrolment, experienced a relapse of symptoms, and deteriorated and died, probably from heart disease, 3 mo after he received the last dose of vaccine. This event was regarded as unrelated to vaccination. There were no other serious adverse events. Adverse events were rare after first and second doses and were not increased in the DNA ME-TRAP group compared to the rabies vaccine group (data not shown). Injection site pain, limited arm motion, headache, and malaise in the first 24 h after vaccination (mild to moderate in intensity, i.e., not preventing activities of daily living, in all but one volunteer) were more common after MVA ME-TRAP vaccination than rabies vaccination (Table 2). Some volunteers developed an injection site blister 1–2 d after MVA ME-TRAP vaccination, which healed over 1–3 wk without complications. Induration (for 1–2 d) and discolouration (faint, shiny macular appearance for several weeks) were common after MVA ME-TRAP vaccination. The frequency of short duration severe adverse events (i.e., preventing activities of daily living) of less than 2% seen with MVA ME-TRAP immunisations is less than that seen with some other licensed alum-formulated vaccines in widespread use.

**Table 2:**
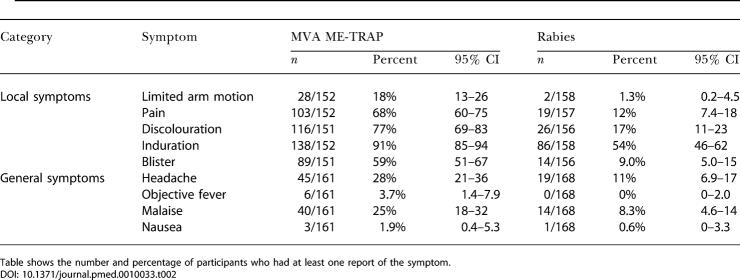
Frequency of Solicited Symptoms during the 7 d after the Third Dose of Vaccine

Table shows the number and percentage of participants who had at least one report of the symptom

### Immunogenicity

In the study, 63 and 30 volunteers from the malaria and rabies vaccine groups, respectively, were assayed 7 d after final vaccination for T cell responses. In the rabies vaccine group, geometric mean effector T cell responses were 3.1, 3.9, and 1.4 spot-forming cells (SFCs) per million PBMCs for T9/96 and 3D7 strains of TRAP and the ME string, respectively. In the malaria vaccine group, the effector T cell frequency to the vaccine strain of the TRAP antigen, T9/96, was geometric mean 251.1 SFCs per million PBMCs (80-fold increase above control group, *p* <0.001, range 6.25–2148.75). A large cross-reactive T cell response to 3D7 TRAP, a strain with 6% amino acid variance to T9/96, and a weaker response to the ME string were also seen at this timepoint (Table 3).

**Table 3:**
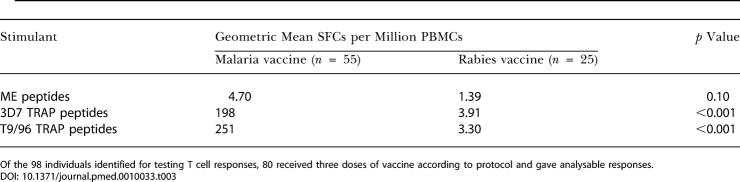
Effector T Cell Responses 1 wk after the Third Vaccination

Of the 98 individuals identified for testing T cell responses, 80 received three doses of vaccine according to protocol and gave analysable responses

### Time to First P. falciparum Infection

**Figure 2:**
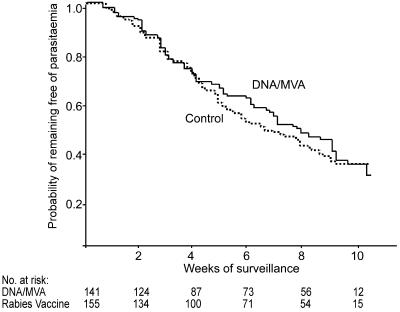
Kaplan-Meier Survival Curves Showing the Probability of Remaining Free of P. falciparum Infection during the 11 wk of Surveillance Week 0 of surveillance began in October 2002, 14 d after the third dose of vaccine was administered.

By the end of the study, 171 participants developed parasitaemia, 80/141 (57%) in the malaria vaccine group and 91/155 (59%) in the rabies vaccine group. The distribution of time to first infection was similar in the two groups (Figure 2). Vaccine efficacy among participants who received three doses, adjusted for age, bednet use, ethnic group, and village of residence was 10.3% (95% CI, −22% to +34%; *p* = 0.49). Similar results were obtained when all participants who received at least one dose of vaccine were included in the analysis (efficacy, 1.0%; 95% CI, −32% to +25%; *p* = 0.95).

Geometric mean P. falciparum densities in first infections were similar in the two groups (31 parasites/μl [IQR, 5–154] in the malaria vaccine group compared to 24 parasites/μl [IQR, 5–69] in the rabies group; Mann-Whitney test, *p* = 0.79).

During surveillance, there were ten episodes of symptomatic malaria in the malaria vaccine group and 13 in the rabies group. The risk of malaria-related symptoms during an episode of parasitaemia was similar in both vaccine groups. Percentage mean packed cell volume at the end of the trial was similar in both groups (41 [IQR, 37–44] for the malaria vaccine group and 40 [IQR, 37–43] for the rabies group).

Within the ELISPOT substudy group, the risk of developing parasitaemia was not associated with effector T cell response to the 3D7 strain of TRAP. The 80 men from the substudy group who received three doses of either malaria vaccine (55 men) or rabies vaccine (25 men), completed 11 wk of surveillance, and had complete ELISPOT data after dose three were divided into four quartiles. Men with the highest effector T cell responses had hazard ratios for infection similar to those with the lowest after adjustment for age, bednet use, and village of residence.

The incidence of parasitaemia decreased with increasing age, and was decreased in those of Fula ethnicity compared to those in the Mandinka and Wollof ethnic groups (Table 4). The use of bednets was not associated with a significantly reduced risk of malaria infection (Table 4).

**Table 4:**
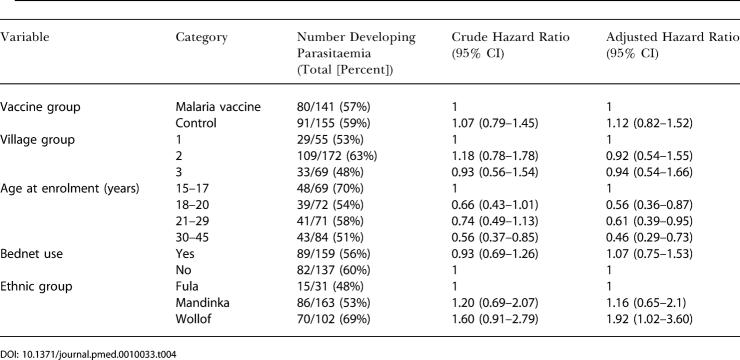
Results of Cox Proportional Hazards Regression Analysis for the Risk of Developing Parasitaemia after Three Doses of Vaccine

## Discussion

This trial demonstrated that vaccination with two doses of DNA ME-TRAP followed by a single dose of MVA ME-TRAP is safe and highly immunogenic for effector T cell induction but that it did not reduce the P. falciparum infection rate in a semi-immune adult African population. This provides a second comparison between protection in malaria-naïve and malaria-experienced adults. RTS,S/AS02, a circumsporozoite protein malaria vaccine based on a hepatitis B surface antigen virus-like particle formulated in a proprietary adjuvant, provided about 40% sterile protection in the artificial challenge model [29] and 71% short-term protection against natural infection [30]. The lack of field efficacy found in the present study despite evidence of partial protection in United Kingdom volunteers supports the use of complete, not partial, protection in the sporozoite challenge model as a predictor of likely field efficacy against malaria infection when screening pre-erythrocytic vaccine candidates. However, some vaccines are known to prevent disease but not infection, as is also the case for naturally acquired immunity to malaria. There was no effect of bednet use on parasitaemia in either this study or an earlier malaria vaccine trial in adults [30], whereas bednet use has been found to substantially reduce the incidence of clinical malaria and childhood mortality in Gambian children [31]. The present study does not exclude the possibility that the vaccination regimen tested could provide significant anti-disease immunity. Paediatric study designs are necessary to evaluate this possibility. The present study highlights an issue related to use of surrogate efficacy endpoints; whereas positive results can spur development, negative results may incorrectly lead to the cessation of development of a candidate vaccine.

Another possible reason for the observed low efficacy is that the frequency of the effector T cell response declines from 7 d after boost, and so efficacy would be prevented if very high frequencies of circulating effectors are needed for protective efficacy. Alternatively, a suboptimal regional memory T cell pool in the liver may be responsible [32], or, less likely in view of the observed T cell strain cross-reactivity, TRAP polymorphisms may have impaired T cell recognition.

In previous studies in East and West Africa, summed T cell responses to TRAP in unvaccinated semi-immune adults by ex vivo γ-interferon ELISPOT were geometric mean less than 20 SFCs per million PBMCs. The candidate regimen represents a new method for induction of unprecedented effector T cell frequencies, which are about 50-fold higher than those induced by lifelong natural exposure. Estimates of the reduction in liver-stage parasite burden induced by these vaccines in the human challenge model are of the order of 80%–90% of infected hepatocytes [21,33]. It is unclear whether a similar level of anti-parasite activity could have been achieved in this study without any significant change in infection rate. Another candidate malaria vaccine that reduced liver parasite burden by an estimated 95% in challenge studies [29,33] did have a substantial, if short-lived, impact on infection rates in a similar Gambian field study [30]. This suggests that a moderate increase in the efficacy of this first-generation prime-boost vaccination strategy in reducing liver parasite burden might have an important impact on overall efficacy.

Second-generation prime-boost vaccine strategies for malaria currently in or near to clinical evaluation include the following: use of a different viral vector as the priming agent that may lead to proportionately greater CD8+ rather than CD4+ T cell induction (J. M. Vuola, S. Keating, D. P. Webster, T. Berthoud, S. Dunachie, et al., unpublished data), as is the case with fowlpox-MVA immunisation; the use of a different antigen, the circumsporozoite protein, or polyprotein constructs [34] to address the difficult issue of target antigen selection; and evaluation of regimes that seek to combine high-level T cell responses with strong anti-sporozoite antibody induction, e.g., protein/adjuvant and recombinant virus prime-boost immunisation. In the medium term, combination with protective blood-stage antigens is also desirable. Determining methods for the successful combination of different candidate vaccine regimens (whether within or between parasite stages) will be one of the important challenges of coming years.

We were unable to obtain a useful estimate of the likely efficacy of the DNA ME-TRAP/MVA ME-TRAP vaccination regime against clinical disease. Even for an adult population, the incidence of clinical disease was lower than expected. Sulphadoxine/pyrimethamine was administered 4 wk before the start of surveillance in this study and in an RTS,S field efficacy study [30]. There is some evidence that pretreatment with this antimalarial reduces the incidence of clinical malaria for longer than 4 wk [35,36]. However, there was also less clinical disease than in recent years in paediatric cohorts recruited for other studies in 2002 at the study site, probably for climatic reasons.

This study highlights North Bank Division in Gambia as an excellent malaria vaccine field trial site both for adults and, by extrapolation, for children. In a low-transmission year, cumulative incidence overall in men aged 15–45 y was 72% over 11 wk, which was higher than expected. Also, compliance was good despite a demanding study design, and migration from the study area was acceptably low.

This paper adds to the body of data detailing the very gradual acquisition of anti-infection immunity in adults resident in sub-Saharan Africa [30]. While substantial immunity to severe malaria is acquired after only a few infections and anti-disease immunity is acquired in childhood, we saw statistically significant decreases in incidence of infection with increasing age in the 15–45 age range (Table 4). The protection against infection for those with Fula ethnicity observed in this trial is consistent with a report from Burkina Faso [37]. The Fulani mostly reside in distinct villages in this part of Gambia.

Immunological analysis of the high level of protection inducible by immunisation of humans and animals with irradiated sporozoites has encouraged attempts to generate protective immunity by subunit vaccines that induce strong cellular immune responses. To date the induction of high-level protective T cell responses against malaria and some other infectious pathogens has generally required two-component prime-boost vaccination approaches [38]. We report the first field efficacy trial of a subunit vaccine designed to induce protective immunity through effector T cell rather than antibody induction. Effector T cell induction 50-fold greater than that generated by natural malaria infection is now possible through DNA-based heterologous prime-boost vaccination of humans. However, further development of T cell–inducing vaccines will be required to evaluate the effects of altering the characteristics, target antigen specificities, and durability of the induced T cells in order to generate higher levels of protective immunity against malaria.

**Patient Summary**

**Background**

Malaria kills 1–2 million people a year, mostly children under the age of five who live in sub-Saharan Africa. Scientists are trying to develop cheap, safe, and effective vaccines that could be given to people living in regions where malaria is very common to prevent them from developing the disease.

**What Did the Researchers Find?**

The researchers enrolled 372 Gambian men aged 15–45 years into the study. They injected half the men with two malaria vaccines, one after the other, and half the men with a rabies vaccine that does not protect against malaria (this vaccine was given so that “control” participants would have some benefit from being in the trial) just before the rainy season, when malaria is especially prevalent. The scientists took blood smears from the men once a week and checked to see if they had been infected with the parasite that causes malaria. They found that the men who had been vaccinated became infected just as quickly as those who had not. Although the two malaria vaccines in concert did not work, neither did they cause any serious side effects. The men given the malaria vaccines did produce an immune response to the vaccines, though not one that was clinically useful.

**What Does This Mean for Patients?**

It looks as though the combination of these two vaccines is not effective at preventing infection with *Plasmodium falciparum,* the parasite that causes malaria. However, there are other vaccines in development that have not been tested yet.

**Resources on the Web.**

Gates Malaria Partnership (which co-funded the study): http://www.lshtm.ac.uk/gmp/

Malaria Vaccine Initiative: http://www.malariavaccine.org/

Medicines for Malaria Venture: http://www.mmv.org/pages/page_main.htm

Roll Back Malaria Partnership: http://rbm.who.int/partnership

The Wellcome Trust (which co-funded the study): http://www.wellcome.ac.uk/en/malaria/

## Supporting Information

### Trial Registration

This trial has been submitted for registration in the International Standard Randomised Controlled Trial Number (ISRCTN) Register. The ISRCTN is ISRCTN05221133; Web site http://www.controlled-trials.com/isrctn/trial/1/0/05221133.html.

Table S1 Consort Checklist (55 KB DOC).

## Abbreviations

ALT: alanine aminotransferase
CI: confidence interval
ELISPOT: enzyme-linked immunospot
IQR: interquartile range
ME: multiple epitope
MVA: modified vaccinia virus Ankara
PBMC: peripheral blood mononuclear cell
SFC: spot-forming cell
TRAP: thrombospondin-related adhesion protein
