# Supplementary material for: A Randomised, Double-Blind, Controlled Vaccine Efficacy Trial of DNA/MVA ME-TRAP Against Malaria Infection in Gambian Adults
Source: PLoS Med. 2004 Oct 26;1(2):e33. doi: 10.1371/journal.pmed.0010033 (PMC524376; doi:10.1371/journal.pmed.0010033)
Supplement: Table S1 — (55 KB DOC). [file pmed.0010033.st001.doc]

| **CONSORT TABLE – MOORTHY et al. JUL 2004 MALARIA VACCINE FIELD TRIAL** | **Item number** | **Descriptor** | **Reported in section:** |
| --- | --- | --- | --- |
| **Title and abstract** | 1 | How participants were allocated to interventions (eg, "random allocation", "randomised", or "randomly assigned"). | Title |
| **Introduction** |  |  |  |
| Background | 2 | Scientific background and explanation of rationale. | Introduction |
| **Methods** |  |  |  |
| Participants | 3 | Eligibility criteria for participants and the settings and locations where the data were collected. | Methods, subsection  Study Setting and Volunteers  paragraph 2,3 |
| Interventions | 4 | Precise details of the interventions intended for each group and how and when they were actually administered. | Methods, subsection  Procedures  Paragraph1,2 |
| Objectives | 5 | Specific objectives and hypotheses. | Introduction para 4 |
| Outcomes | 6 | Clearly defined primary and secondary outcome measures and, when applicable, any methods used to enhance the quality of measurements (eg, multiple observations, training of assessors, &c). | Methods, subsection  Statistical analysis para 1 |
| Sample size | 7 | How sample size was determined and, when applicable, explanation of any interim analyses and stopping rules. | Methods, subsection  Study Setting and Volunteers , para 2 |
| **Randomisation** |  |  |  |
| Sequence generation | 8 | Method used to generate the random allocation sequence, including details of any restriction (eg, blocking, stratification). | Methods, subsection  Procedures  Paragraph1, |
| Allocation concealment | 9 | Method used to implement the random allocation sequence (eg, numbered containers or central telephone), clarifying whether the sequence was concealed until interventions were assigned. | Methods, subsection  Procedures  Paragraph1 |
| Implementation | 10 | Who generated the allocation sequence, who enrolled participants, and who assigned participants to their groups. | Methods, subsection  Procedures  Paragraph1 |
| Blinding (masking) | 11 | Whether or not participants, those administering the interventions, and those assessing the outcomes were aware of group assignment. If not, how the success of masking was assessed. | Methods, subsection  Procedures  Paragraph1 |
| Statistical methods | 12 | Statistical methods used to compare groups for primary outcome(s); methods for additional analyses, such as subgroup analyses and adjusted analyses. | Methods, subsection  Statistical analysis para 1 |

| **Results** |  |  |  |
| --- | --- | --- | --- |
| Participant flow | 13 | Flow of participants through each stage (a diagram is strongly recommended). Specifically, for each group, report the numbers of participants randomly assigned, receiving intended treatment, completing the study protocol, and analysed for the primary outcome. Describe protocol deviations from study as planned, together with reasons. | Fig 1 |
| Recruitment | 14 | Dates defining the periods of recruitment and follow-up. | 5,8 |
| Baseline data | 15 | Baseline demographic and clinical characteristics of each group. | Table 1 |
| Numbers analysed | 16 | Number of participants (denominator) in each group included in each analysis and whether the analysis was by "intention to treat". State the results in absolute numbers when feasible (eg, 10/20, not 50%). | Results, para 1 |
| Outcomes and estimation | 17 | For each primary and secondary outcome, a summary of results for each group, and the estimated effect size and its precision (eg, 95% CI). | Results, subsections Time to First P. falciparum Infection and Effector T cell induction |
| Ancillary analyses | 18 | Address multiplicity by reporting any other analyses performed, including subgroup analyses and adjusted analyses, indicating those prespecified and those exploratory. | Results, all subsections |
| Adverse events | 19 | All important adverse events or side-effects in each intervention group. | Results, subsection Adverse events, Table 2 |
| **Discussion** |  |  |  |
| Interpretation | 20 | Interpretation of the results, taking into account study hypotheses, sources of potential bias or imprecision and the dangers associated with multiplicity of analyses and outcomes. | Discussion |
| Generalisability | 21 | Generalisability (external validity) of the trial findings. | Discussion |
| Overall evidence | 22 | General interpretation of the results in the context of current evidence. | Discussion |

**Checklist of items to include when reporting a randomised trial**
